# Impact of Influenza Vaccination on Mortality and Major Cardiovascular Events in Adults with Cardiovascular Disease: A Systematic Review and Meta-Analysis of Randomized Controlled Trials

**DOI:** 10.3390/vaccines14040309

**Published:** 2026-03-29

**Authors:** Sitah Alotaibi, Mazen Bostaji, Anas Alharbi, Latifa Alaqeel, Ghaida Alsharif, Afaf Albalawi, Naif Aloufi, Khaled Khormi, Wedad Almarhbi, Seham Abulkhair, Turki Alshaikh, Abdullah Almaqhawi

**Affiliations:** 1 Faculty of Nursing, Princess Nourah Bint Abdulrahman University, Riyadh 11671, Saudi Arabia; 2 Faculty of Medicine, Umm Al Qura University, Makkah 21955, Saudi Arabia; s442004518@uqu.edu.sa; 3 Family Medicine Department, Ministry of National Guard Health Affairs, Riyadh 11671, Saudi Arabia; alharbian2@mngha.med.sa; 4 Faculty of Medicine, Imam Mohammad Ibn Saud Islamic University, Riyadh 11671, Saudi Arabia; 442022274@sm.imamu.edu.sa; 5 Faculty of Medicine, University of Jeddah, Jeddah 21589, Saudi Arabia; 2211247@uj.edu.sa; 6 Family Medicine Specialist General Administration of Medical Services, Tabuk University, Tabuk 71491, Saudi Arabia; afa_albalawi@ut.edu.sa; 7 Faculty of Medicine, Qassim University, Buraidah 51452, Saudi Arabia; 411116480@qu.edu.sa; 8 Prince Sultan Military Medical City, Riyadh 11671, Saudi Arabia; up.khaledjubran@gmail.com; 9 Faculty of Medicine, Umm Al Qura University, Al Qunfudhah 24381, Saudi Arabia; s439003883@uqu.edu.sa; 10 Microbiology, Faculty of Science, Jeddah Unifigureversity, Jeddah 21959, Saudi Arabia; 1825383@uj.edu.sa; 11 King Abdullah Medical Complex, Jeddah 21577, Saudi Arabia; ttalshaikh@moh.gov.sa; 12 Department of Family and Community Medicine, College of Medicine, King Faisal University, Al Ahsa P.O. Box 400, Saudi Arabia; aalmuqahwi@kfu.edu.sa

**Keywords:** influenza vaccination, cardiovascular disease, major adverse cardiovascular events, mortality, randomized controlled trials

## Abstract

**Background**: Cardiovascular disease (CVD) is a leading cause of morbidity and mortality worldwide, and seasonal influenza is a recognized trigger of acute cardiovascular events. The influenza vaccine has been proposed as a secondary preventative measure, though the benefits regarding mortality and cardiovascular events are unclear. **Objective**: This study aimed to establish the efficacy of influenza vaccination in lowering mortality and major cardiovascular events in adults suffering from established CVD. **Methods**: A systematic review and meta-analysis of randomized controlled trials (RCTs) was conducted. Eligible studies were identified from PubMed, Google Scholar, and The Cochrane Central Register of Controlled Trials (CENTRAL) through November 2025. RCTs involving adults with established CVD evaluating influenza vaccine impact in comparison to placebo or standard care were included. The Cochrane Risk of Bias tool 2 was used to evaluate study quality. **Results**: A total of eight RCTs met the inclusion criteria. Influenza vaccination was associated with a statistically significant decrease in the composite outcome (of all-cause mortality, acute myocardial infarction, and stent thrombosis) (OR 0.71, 95% CI 0.57–0.90; *p* = 0.004; I^2^ = 0%) and major adverse cardiovascular events (OR 0.44, 95% CI 0.26–0.74; *p* = 0.002; I^2^ = 0%). Cardiovascular mortality was found to be significantly lower in the vaccination group (OR 0.64, 95% CI 0.47–0.86; *p* = 0.003). However, all-cause mortality was not significantly reduced (OR 1.13, 95% CI 0.79–1.62; *p* = 0.50; I^2^ = 88%). **Conclusions**: Influenza vaccination was associated with a reduction in combined cardiovascular events and mortality due to cardiovascular causes in adults diagnosed with established CVD. In contrast, all-cause mortality was not significantly reduced, and the evidence for this outcome was inconclusive. This supports the routine use of the vaccine as an adjunctive measure in the secondary prevention of CVD.

## 1. Introduction

Cardiovascular disease (CVD) is the leading cause of morbidity and mortality worldwide, accounting for over 18 million annual deaths despite major advances in the management of the condition [1]. Despite optimal management of secondary prevention, the incidences of recurrent acute coronary heart disease, stroke, decompensated heart failure, or cardiovascular-related death remain unacceptably high [2].

The influenza virus has been identified as a trigger of acute episodes of cardiovascular diseases [3]. Several large-scale studies have shown a reverse correlation between influenza vaccination and fatal and nonfatal episodes of cardiac events in individuals at high cardiac risk [4]. This correlation has a biological rationale, since influenza virus infection leads to acute inflammation and hypercoagulable state, which are accompanied by a brief impairment of endothelial dysfunction and accelerate atherosclerotic plaque instability [5]. Seasonal influenza remains a major global cause of acute respiratory illness, with an estimated 1 billion infections annually, including 3–5 million severe cases and 290,000–650,000 respiratory deaths [6]. In adults, influenza accounts for approximately 5–10% of infections, while in children it accounts for 20–30% [7]. More broadly, vaccination is increasingly recognized as a preventive strategy with potential cardiovascular relevance in several high-risk populations, including older adults, patients with heart failure or congenital heart disease, pregnant women with cardiovascular conditions, and immunocompromised patients such as heart transplant recipients [8,9].

Based on such mechanisms, the influenza vaccine has been proposed to represent a cardioprotective strategy in those at increased cardiovascular risk [10]. Randomized controlled trials performed in patients with acute coronary syndrome have shown a reduction in cardiovascular death and composite cardiovascular events after influenza vaccination [11]. The Influenza Vaccination After Myocardial Infarction (IAMI) trial indicated there was a greatly reduced chance of all-cause and cardiovascular death after early influenza vaccination post myocardial infarction compared with late vaccination [12]. A reduction in pre-specified ischemic and composite cardiovascular events after influenza vaccination was observed among others in additional randomized controlled trials: The Influenza Vaccination After Myocardial Infarction trial, FLUVACS, and FLUCAD [13,14,15].

Randomized-trial data in heart failure patients suggest a potential beneficial effect of influenza vaccination on the heart and blood vessels, as seen in the multicountry PANDA II trial, which showed trends toward a positive outcome in vaccine recipients compared with unvaccinated patients [16]. Some recent studies have explored whether high-dose influenza vaccination may be superior to standard-dose formulations in selected populations, although the evidence remains limited [17]. While large-scale research studies have found correlations between influenza vaccination and a reduced risk of all-cause and CV-related death, RCTs are required to confirm an association and provide specific trial data to guide treatment recommendations [18].

Despite the existence of RCTs, however, the current evidence base does not provide sufficient power to conclude definitively about the effect of influenza vaccination on cardiovascular deaths and major adverse cardiovascular events [19]. This is because trial data currently consist of studies that have examined diverse populations and outcome measures. Additionally, this data has been aggregated based on data from both RCT and observational studies in previously conducted meta-analyses, which have, in effect, limited the power to conclusively state a protective effect of vaccination on cardiovascular disease [20,21].

The aim of this systematic review and meta-analysis is to determine whether inactivated seasonal influenza vaccines reduce all-cause mortality and serious cardiovascular adverse events in adults with cardiovascular disease. Drawing on exclusively randomized-controlled-trial data, this paper seeks to provide high-quality, actionable information for patients and physicians alike on cardiovascular disease prevention strategies.

## 2. Materials and Methods

### 2.1. Study Protocol and Reporting Guidelines

The present systematic review and meta-analysis was carried out in line with the Preferred Reporting Items for Systematic Reviews and Meta-Analyses (PRISMA) 2020 guidelines [22]. An a priori protocol of this study was formulated and was prospectively registered with the International Prospective Register of Systematic Reviews (PROSPERO) under registration ID CRD420251175356.

### 2.2. Search Strategy

An extensive literature search was conducted across PubMed/MEDLINE, the Cochrane Central Register of Controlled Trials (CENTRAL), and Google Scholar from their inception to November 2025. Controlled vocabulary and free-text terms related to influenza vaccination and cardiovascular outcomes were connected using Boolean operators to formulate the following search strategy: [(“influenza vaccine” OR “influenza vaccination” OR “flu shot”) AND (“cardiovascular disease” OR “coronary artery disease” OR “myocardial infarction” OR “heart failure” OR “stroke” OR “major adverse cardiovascular events” OR mortality OR hospitalization)]. The search strategy was edited according to each database. In addition, terms to identify randomized controlled trials were included. Finally, reference lists of eligible studies and relevant reviews were manually screened to identify additional trials.

### 2.3. Eligibility Criteria

The criteria for the studies being considered were carefully described. Trials were considered for inclusion if they were randomized controlled trials involving participants with established coronary heart disease or other forms of atherosclerotic vascular disease and who were 18 or older. In addition, the trials had to involve the inactivated influenza vaccine administered annually. Studies had to report at least one cardiovascular outcome of interest (all-cause mortality, cardiovascular mortality, myocardial infarction, or major adverse cardiovascular events) and have a minimum follow-up of 12 months. There were no language restrictions. Studies were excluded if they enrolled mainly pediatric populations, had fewer than 50% of participants with cardiovascular disease at baseline, used non-influenza vaccines or non-standard regimens, lacked a comparator group, did not report extractable outcomes, or were non-randomized designs such as observational or single-arm studies.

### 2.4. Screening and Data Extraction

Titles and abstracts of identified records were independently screened by two reviewers using Rayyan AI, with discrepancies resolved through discussion and consensus. Subsequently, full-text articles were independently assessed by two more reviewers, and any disagreements were resolved by a third reviewer. Following this, data extraction was performed using a standardized form by an independent, contracted statistical team. Extracted data included study characteristics, sample size, baseline demographics, intervention and comparator details, follow-up duration, and outcome event data for each study arm, including major adverse cardiovascular events, myocardial infarction (ST-segment elevation and non-ST-segment elevation where reported), cardiovascular mortality, all-cause mortality, and the predefined composite outcome (defined as trial-reported endpoints consisting of two or more prespecified cardiovascular events, where occurrence of any component counted as an event; these were extracted according to the original trial definitions without post hoc reconstruction or harmonization).

### 2.5. Risk-of-Bias and Garde Assessment

To ensure methodological quality, two reviewers independently evaluated the bias of included randomized controlled trials using the Cochrane Risk of Bias tool for randomized trials, with disagreements resolved by discussion and consensus. To formally assess the certainty of the evidence presented in this meta-analysis, we applied the Grading of Recommendations Assessment, Development and Evaluation (GRADE) framework. The certainty of evidence for the composite endpoint of all-cause death, acute myocardial infarction, or stent thrombosis was rated as moderate. Similarly, the evidence for a reduction in major adverse cardiovascular events (MACEs) and cardiovascular mortality was moderate. This rating reflects that while the evidence is derived from randomized controlled trials, the overall certainty was downgraded due to the potential for risk of bias across the included studies.

In contrast, the certainty of evidence for a reduction in myocardial infarction as a standalone outcome was rated as low, due to both the risk of bias and imprecision in the effect estimate. Furthermore, the evidence for an effect on all-cause mortality was rated as very low. This was due to concerns about risk of bias, significant and unexplained inconsistency between the studies, and statistical imprecision. This formal assessment underscores that while the influenza vaccine shows a clear benefit for several important cardiovascular outcomes, the evidence supporting a reduction in all-cause mortality remains inconclusive.

### 2.6. Data Synthesis and Statistical Analysis

Quantitative synthesis was carried out with the Cochrane Review Manager (RevMan) Version 5.3 [23]. For the dichotomous outcomes, the pooled odds ratios with their corresponding 95% confidence intervals were calculated. OR was selected a priori as a standard relative effect measure for binary outcomes in meta-analysis because it is mathematically symmetric with respect to event/non-event definition and can be less sensitive to variation in baseline risk across studies than the risk ratio. Because the analyses were performed in RevMan 5.3, the Hartung–Knapp adjustment was not applied in the present meta-analysis. The random-effect model using the DerSimonian–Laird approach was used to account for possible variability in the outcome measures of the studies. The statistical heterogeneity was assessed by Cochran’s Q test, Tau^2^, and the I^2^ test and judged according to the Cochrane Handbook with the following levels: 0–40% for low heterogeneity, 30–60% for moderate heterogeneity, and 50–90% for substantial heterogeneity. Sensitivity analyses examined the stability of the results from the random-effect model using the leave-one-out technique. Funnel plots were analyzed to check for the possibility of publication bias. Also, we assessed the certainty of evidence for each outcome using the GRADE (Grading of Recommendations Assessment, Development and Evaluation) framework by considering the domains of risk of bias, inconsistency, indirectness, imprecision, and publication bias.

## 3. Results

### 3.1. Study Selection

**Figure 1:**
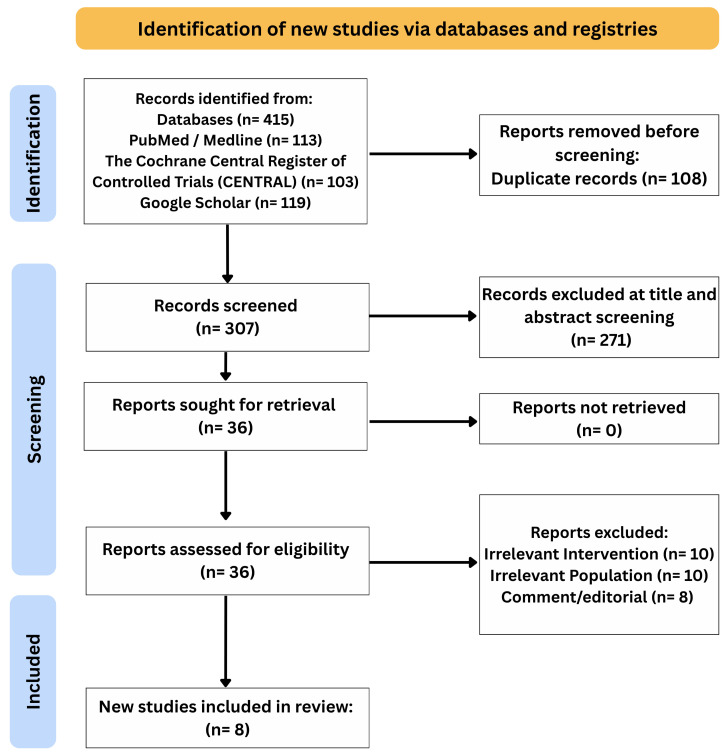
PRISMA chart of the included studies.

The comprehensive search across PubMed, Cochrane, and Google Scholar databases yielded the identification of 415 records. After excluding duplicates, 307 records were screened based on titles and abstracts, resulting in the exclusion of 271 articles. Subsequently, 36 full-text articles were assessed against the previously specified eligibility criteria, after which 28 articles were excluded based on pre-specified criteria. Finally, eight studies were included in the systematic review after inclusion criteria were met (Figure 1).

### 3.2. Characteristics of Included Studies

Eight articles comparing the efficacy and safety of the influenza vaccine versus control were included in our study: Gurfinkel et al. (FLUVACS) [14], Ciszewski et al. (FLUCAD) [15], Phrommintikul et al. [11], Fröbert et al. (IAMI) [13], Akhtar et al. (IAMI trial analysis) [12], Loeb et al. [16], Dehesh et al. [24], and Anderson et al. [25]. All included studies were randomized controlled trials. The studies sample ranged from 200 to 7771 patients. Most studies were conducted in the United States, the United Kingdom, Italy, Germany, Japan, and South Korea. The mean age of participants ranged from 54.5 years to 71 years. The summary characteristics of the included studies are reported in Table 1. The baseline characteristics of the included studies are shown in Table 2.

### 3.3. Risk-of-Bias Assessment

**Figure 2:**
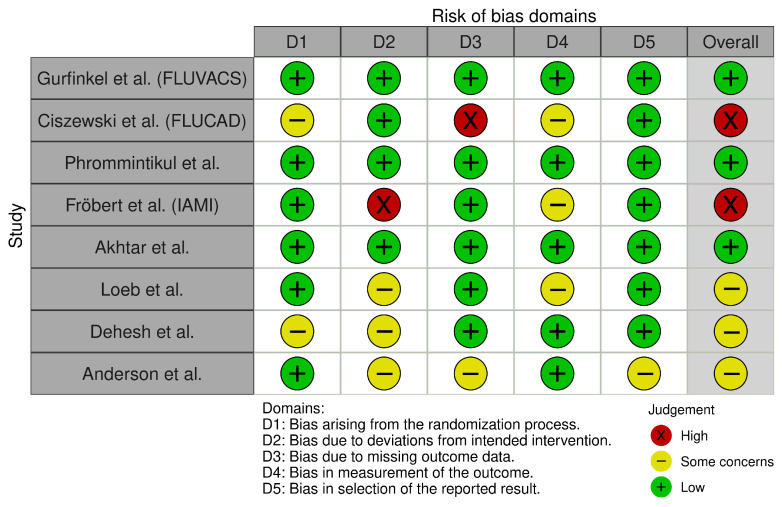
Risk-of-bias assessment of the included studies using the RoB 2 tool. Included studies were Phrommintikul et al. [11], Akhtar et al. [12], Fröbert et al. (IAMI) [13], Gurfinkel et al. (FLUVACS) [14], Ciszewski et al. (FLUCAD) [15], Loeb et al. [16], Dehesh et al. [24], and Anderson et al. [25].

Overall, the risk of bias was low to moderate across the included studies. Three studies were judged as low risk, three had some concerns, and two were rated as high risk of bias. The domains in which concerns or high risk were most commonly identified were deviations from intended interventions and missing outcome data (Figure 2).

### 3.4. Primary Outcomes

#### 3.4.1. The Composite of All-Cause Death, AMI, or Stent Thrombosis

**Figure 3:**
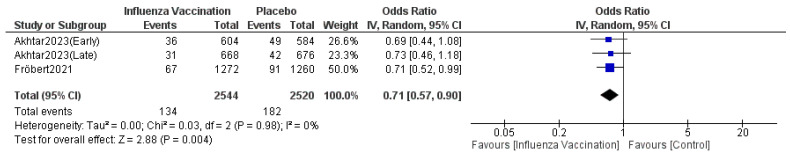
Forest plot of the composite of all-cause death, AMI, or stent thrombosis. Included studies were Akhtar et al. [12] and Fröbert et al. [13].

After outcome result extraction of three trials, data were pooled. The pooled results indicated that the odds ratio for the composite of all-cause death, AMI, or stent thrombosis is 0.71 (0.57, 0.90), with a 95% CI favoring influenza vaccination compared to the control. The results were statistically significant (*p* = 0.004) (Figure 3). The sensitivity analysis revealed no heterogeneity (I^2^ = 0%).

#### 3.4.2. MACEs: Cardiovascular Death, MI, or Coronary Revascularization

**Figure 4:**
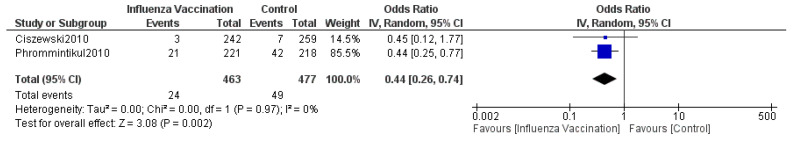
Forest plot of MACEs: cardiovascular death, MI, or coronary revascularization. Included studies were Phrommintikul et al. [11] and Ciszewski et al. [15].

The pooled results indicated that the odds ratio for MACEs (cardiovascular death, MI, or coronary revascularization) is 0.44 (0.26, 0.74), with a 95% CI favoring influenza vaccination compared to the control. The results were statistically significant (*p* = 0.002) (Figure 4). The sensitivity analysis revealed no heterogeneity (I^2^ = 0%).

#### 3.4.3. Myocardial Infarction

**Figure 5:**
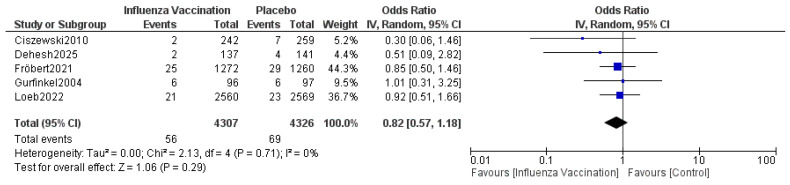
Forest plot of myocardial infarction. Included studies were Fröbert et al. [13], Gurfinkel et al. [14], Ciszewski et al. [15], Loeb et al. [16] and Dehesh et al. [24].

**Figure 6:**
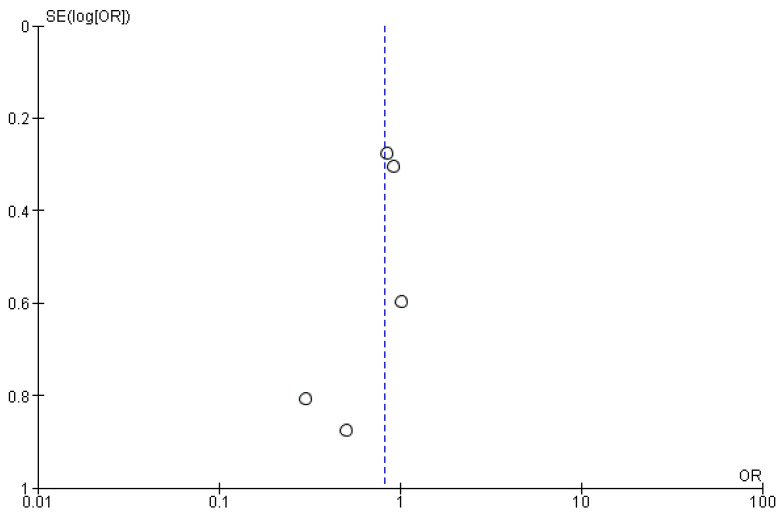
Funnel plot of myocardial infarction. Open circles represent individual studies, plotted according to effect size (OR) on the x-axis and standard error of the log odds ratio, SE(log[OR]), on the y-axis. The dashed vertical line indicates the pooled effect estimate.

The pooled results indicated that the odds ratio of myocardial infarction is 0.82 (0.57, 1.18) with 95% CI favoring TXA over influenza vaccination compared to the control. The results were not statistically significant (*p* = 0.29) (Figure 5). The sensitivity analysis revealed no heterogeneity (I^2^ = 0%). The funnel plot of myocardial infarction is shown in Figure 6.

#### 3.4.4. Cardiovascular Death

**Figure 7:**
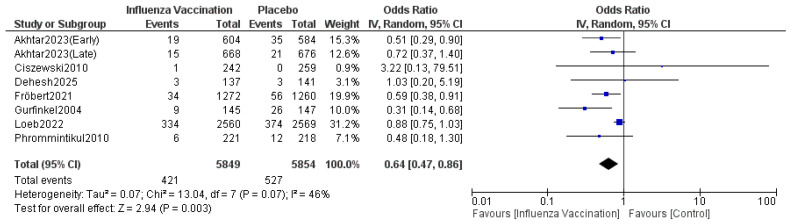
Forest plot of cardiovascular death. Included studies were Phrommintikul et al. [11], Akhtar et al. [12], Fröbert et al. [13], Gurfinkel et al. [14], Ciszewski et al. [15], Loeb et al. [16] and Dehesh et al. [24].

**Figure 8:**
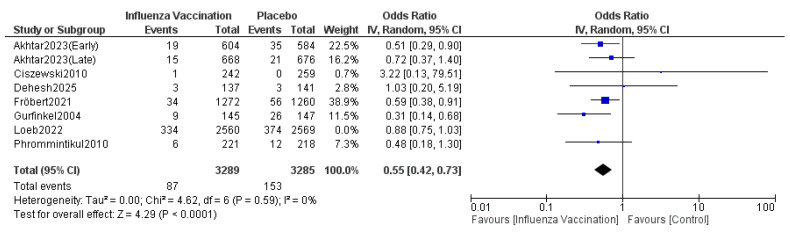
Sensitivity plot of cardiovascular death. Included studies were Phrommintikul et al. [11], Akhtar et al. [12], Fröbert et al. [13], Gurfinkel et al. [14], Ciszewski et al. [15], Loeb et al. [16] and Dehesh et al. [24].

**Figure 9:**
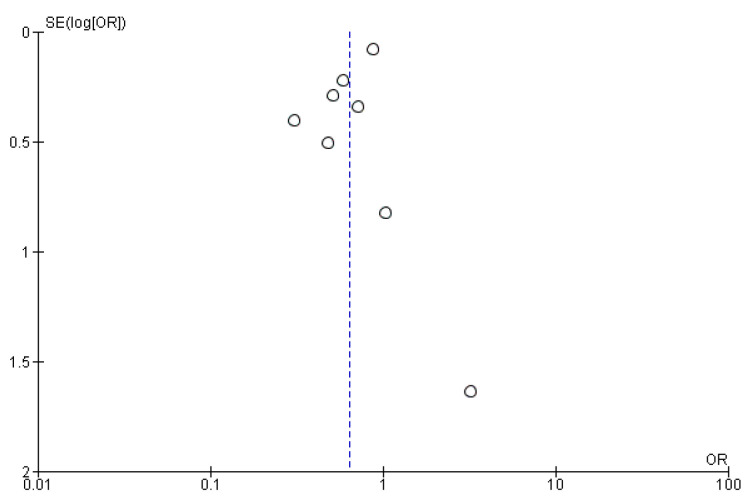
Funnel plot of cardiovascular death. Open circles represent individual studies, plotted according to effect size (OR) on the x-axis and standard error of the log odds ratio, SE(log[OR]), on the y-axis. The dashed vertical line indicates the pooled effect estimate.

The pooled results indicated that the odds ratio for cardiovascular death is 0.64 (0.47, 0.86), with 95% CI favoring TXA over influenza vaccination compared with the control. The results were statistically significant (*p* = 0.003) (Figure 7). The sensitivity analysis revealed moderate heterogeneity (I^2^ = 46%), which was resolved after omitting Loeb 2022 (Figure 8). The funnel plot of cardiovascular death is shown in Figure 9.

#### 3.4.5. All-Cause Deaths

**Figure 10:**
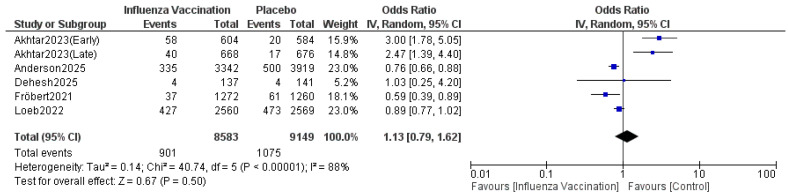
Forest plot of all-cause deaths. Included studies were Akhtar et al. [12], Fröbert et al. [13], Loeb et al. [16], Dehesh et al. [24] and Anderson et al. [25].

**Figure 11:**
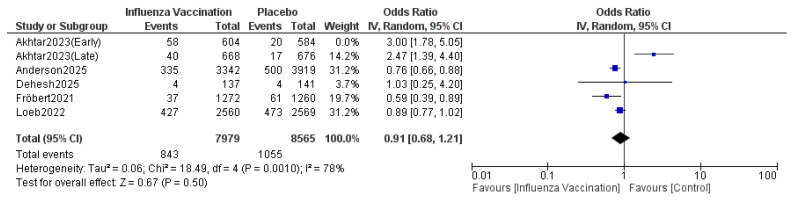
Sensitivity plot of all-cause deaths. Included studies were Akhtar et al. [12], Fröbert et al. [13], Loeb et al. [16], Dehesh et al. [24] and Anderson et al. [25].

**Figure 12:**
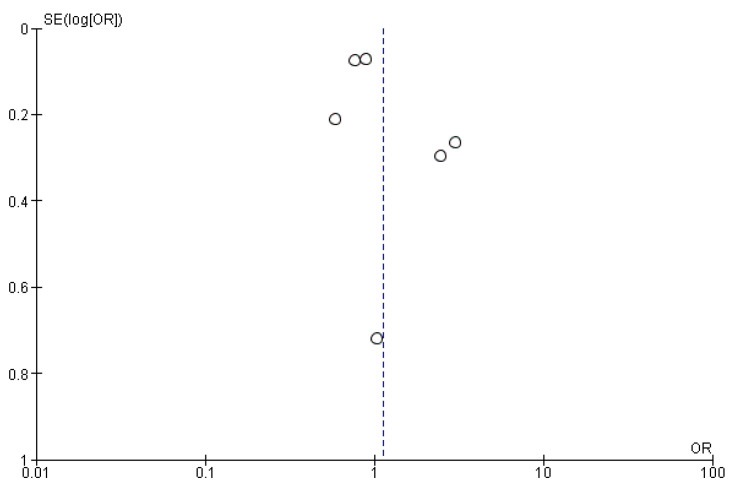
Funnel plot of all-cause deaths. Open circles represent individual studies, plotted according to effect size (OR) on the x-axis and standard error of the log odds ratio, SE(log[OR]), on the y-axis. The dashed vertical line indicates the pooled effect estimate.

The pooled results indicated that the odds ratio of all-cause deaths is 1.13 (0.79, 1.62) with 95% CI favoring TXA compared to influenza vaccination. The results were not statistically significant (*p* = 0.50) (Figure 10). The sensitivity analysis revealed substantial heterogeneity (I^2^ = 88%), which persisted after omitting Akhtar 2023 (Early) (Figure 11). The funnel plot of all-cause deaths is shown in Figure 12.

#### 3.4.6. Certainty of Evidence

Certainty of evidence assessed using the GRADE approach was moderate for the composite of all-cause death, acute myocardial infarction, or stent thrombosis, for major adverse cardiovascular events, and for cardiovascular death, each downgraded by one level for risk of bias. The certainty of evidence for myocardial infarction was low because of the risk of bias and imprecision. In contrast, the certainty of the evidence for all-cause mortality was very low due to risk of bias, substantial inconsistency, and imprecision. Detailed domain-level judgments are provided in Table 3.

## 4. Discussion

### 4.1. Influenza Prevalence

Our meta-analysis suggests that influenza vaccination reduces composite cardiovascular events and cardiovascular mortality in adults with established cardiovascular disease.

Adults comprise approximately 5–10% of infections, and for children, 20–30%, emphasizing how often there is exposure to vulnerable patients with CVD [7]. Influenza is more than just a respiratory illness. It often causes acute CVD events. Seasonal influenza activity is proportional to excess deaths from CVD in older individuals [26]. Infections due to influenza significantly increase CVD risk in vulnerable individuals [27]. Our meta-analysis further supports the notion that influenza vaccination for patients with existing CAD after prior stenting is not only used to control infections but also to modify CVD risk.

CVD remains the leading cause of death worldwide, and myocardial infarction and stroke account for most cardiovascular deaths [28,29]. In this context, our pooled results showing reductions in composite cardiovascular outcomes and cardiovascular mortality suggest that influenza vaccination may be a clinically relevant adjunctive secondary prevention strategy in patients with established cardiovascular disease. Given the substantial global burden of CVD, even a modest relative reduction in recurrent cardiovascular events could translate into meaningful public health benefit, particularly in high-risk populations and in low- and middle-income settings where cardiovascular mortality remains high [24]. Against such a background of major global and regional CVD burden, the potential contribution of any safe and low-cost intervention with proven efficacy in reducing recurrent cardiovascular events, such as vaccination against seasonal influenza, is significant.

We found that influenza vaccination was associated with significant reductions in all-cause death, acute myocardial infarction (AMI), or stent thrombosis (OR 0.71), in MACEs, which are defined as cardiovascular deaths, MIs, or coronary revascularization (OR 0.44), and in cardiovascular deaths (OR 0.64), although there were no statistically significant effects on MIs and all-cause mortality. These results are consistent with the current literature, which continues to show benefits of influenza vaccination among patients with coronary disease or those who recently had acute coronary syndrome (ACS) [30,31,32,33]. In Behrouzi’s updated meta-analysis, influenza vaccination reduced the risk of cardiovascular events by 34% and had a greater effect in patients with recent ACS [30]. Additionally, Jaiswal and a recent meta-analysis of RCTs of ischemic heart disease reported a lower risk of MACEs and cardiovascular death in the vaccinated patients [31]. More broad living-evidence syntheses across heterogeneous cardiovascular populations show that the mortality effect of vaccination is less stable than the composite event effect [32,33], which helps explain why our all-cause mortality result was neutral despite favorable cardiovascular results.

As contextual background, some recent studies have explored whether different influenza vaccine formulations, including high-dose versus standard-dose vaccines, may have differing effects in selected populations. Current evidence remains limited. In the INVESTED randomized trial, high-dose trivalent influenza vaccines did not provide a significant reduction in all-cause mortality or cardiopulmonary hospitalization compared to standard-dose quadrivalent vaccines in high-risk patients with cardiovascular disease [34]. On the contrary, a prespecified analysis of the DANFLU-1 trial reported that high-dose quadrivalent vaccines may reduce hospitalization due to pneumonia or influenza, as well as all-cause mortality in older adults. However, there was no pattern of benefit in the cardiovascular subgroups, so the findings of this study were considered hypothesis-generating [35]. Thus, high-dose influenza vaccines may have some potential in certain older populations, but the evidence does not support the claim that they are more effective than standard-dose vaccines for preventing cardiovascular events. As direct comparisons between influenza vaccine formulations were beyond the scope of this meta-analysis, these data are mentioned only to acknowledge an emerging area of research.

This meta-analysis focuses on adults with cardiovascular disease. However, there are several other cardiovascular-sensitive populations for whom influenza vaccination would be relevant. Patients with congenital heart disease, those with advanced heart failure awaiting transplant, heart transplant recipients, and postoperative cardiovascular surgery patients may be particularly at risk for negative consequences from influenza infections. In these patients, the influenza vaccine may be clinically relevant for cardiovascular disease prevention, although there is less evidence available for these populations than for patients with coronary artery disease or heart failure. Concerns around vaccine-associated myocarditis should be noted here, although it is less relevant to this analysis. There are mostly concerns about the issue with non-influenza vaccines, which are not the focus of the studies of the influenza vaccines included in this analysis. These considerations are important, but are secondary to the main conclusions of this analysis.

More contemporary guidance emerges in the form of the Saudi Heart Association Position Statement on “Adult Cardiovascular Vaccination,” recommending influenza vaccination as a fundamental component of the broad portfolio of cardiovascular medicine, including vaccination against COVID-19, RSV, pneumococcus, and other indicated pathogens, on the basis of the use of vaccination in preventing serious adverse cardiovascular events in persons known to have CVD [36]. However, the policy-making effort might yield little if the current trend of low adoption persists.

### 4.2. Clinical Implications

The trend observed in our outcomes suggests that the influenza vaccine modifies cardiovascular risk for composite and cardiovascular outcomes, rather than a general lowering of all-cause mortality over a short- to medium-term period. Indeed, this can be seen to be consistent with previous meta-analyses, which have shown a 30% to 45% reduced risk of major cardiovascular events and cardiovascular deaths among vaccinated high-risk patients [37].

From an evidence point of view, these study findings support an approach for its use similar to drug treatment as another tool for secondary prevention among individuals with CAD, together with antiplatelet therapy, statins, and renin–angiotensin system antagonists. In Saudi Arabia, where there is significant mortality due to cardiovascular disease and where there is also heightened exposure to flu infection due to Hajj and Umrah pilgrimages, systematic vaccination practices among heart patients can lead to significant absolute reductions in risk, specifically for seniors and diabetic or heart failure patients. From another perspective, these study findings also support maintaining policy leaps based on evidence as statistically validated, but there remains a suboptimal vaccination rate. The need for cardiologists, internists, and family care specialists to prescribe flu vaccination as another aspect of heart care, distinct from its peripheral concern to general public health officials as specialists, arises from this observed need for policy correction to support excluded or under-mandated individuals.

### 4.3. Strengths and Limitations

One of the advantages of the study lies in the specific cardiovascular population the study focused on; all trials included in the meta-analysis evaluated flu vaccines vs. controls in proven CAD and those with PCI and/or stented patients. The analysis used random-effect models, systematically assessed heterogeneity (I^2^), and applied leave-one-out sensitivity analyses to explore influential trials. For most primary outcomes, heterogeneity was low, and the direction of effect was consistent. Funnel plot inspection for key endpoints did not reveal marked asymmetry, suggesting no obvious large publication bias, although this cannot be completely excluded. In particular, the small number of included randomized trials limits the reliability of funnel plot assessment, and it remains possible that unpublished or selectively unreported studies with neutral or unfavorable findings were not captured.

However, several limitations should be acknowledged. First, the number of available randomized trials remains modest, and sample sizes, follow-up durations, and event rates vary considerably across studies. Second, definitions of MACEs and composite endpoints were not fully uniform, and background therapy (e.g., statin use, dual antiplatelet therapy) differed across time periods and geographic settings, potentially influencing absolute event rates. Third, vaccine formulations that match circulating strains and the timing of vaccination relative to the influenza season may have varied, introducing clinical heterogeneity that is not fully captured in pooled estimates. Finally, although our findings may be relevant to other high-risk cardiovascular groups, including patients with congenital heart disease, transplant-related conditions, or recent cardiovascular surgery, these populations were not specifically examined in the included trials and should not be overinterpreted based on the present analysis alone.

The all-cause mortality outcome showed substantial heterogeneity that persisted even after sensitivity analyses, suggesting that this endpoint may be influenced by factors unrelated to influenza or cardiovascular mechanisms (e.g., non-cardiovascular deaths, competing risks, or pandemic-era effects in more recent trials). Finally, most data derive from high- and middle-income settings; direct evidence from Gulf or Saudi populations is limited, and extrapolation to local practice assumes similar vaccine effectiveness and background risk. Observational data and pragmatic trials in Saudi patients with coronary disease, particularly around the Hajj and Umrah seasons, would therefore be valuable.

## 5. Conclusions

The present systematic review and meta-analysis suggests that influenza vaccination may be associated with a reduction in composite cardiovascular outcomes and cardiovascular mortality in adults with established cardiovascular disease. These findings suggest that influenza vaccination may be a practical and potentially beneficial adjunctive strategy in secondary cardiovascular prevention, particularly among patients at elevated cardiovascular risk. Although statistically significant benefits were not consistently observed for all individual endpoints, including all-cause mortality and myocardial infarction, the overall pattern of results generally favors vaccination, although these findings should be interpreted cautiously given the limited number of included studies. Given its availability, safety profile, and potential cardiovascular benefit, influenza vaccination may be considered within comprehensive preventive care for patients with cardiovascular disease. Addressing the disparity between national recommendations and current vaccination practices should be a top priority for cardiology service provision and public health programs. Bridging this gap is critical to optimizing health outcomes while limiting the burden of influenza-related cardiovascular disease. Further large-scale, high-quality randomized controlled trials are needed to confirm these findings and to better clarify influenza vaccination’s effect on specific cardiovascular outcomes.

## Figures and Tables

**Table 1:** The summary characteristics of the included studies.

ID	Country/Setting	Study Design	Intervention	Duration	Results	Conclusion
**Ciszewski 2010** **[15]**	Poland	A prospective, randomized, double-blind, single-center, placebo-controlled study	Influenza vaccine vs. placebo	12 months	• **Study focus:** Patients with coronary artery disease, including acute coronary syndrome (ACS) treated with primary PCI and stable angina. • In ACS patients, vaccination reduced coronary ischemic events (HR 0.37; 95% CI 0.14–0.99; *p* = 0.047). • A trend toward lower MACEs was observed, but it was not statistically significant (HR 0.36; 95% CI 0.09–1.39; *p* = 0.139). • No significant effect on cardiovascular mortality was observed. • No clear benefit was seen in patients with stable angina.	The preventive effect of influenza vaccination on the clinical course of coronary artery disease was observed in patients with acute coronary syndrome, but not in those with stable angina. Influenza immunization can be safely administered soon after acute coronary syndrome and primary PCI.
**Fröbert 2021** **[13]**	30 centers in 8 countries (Sweden, Denmark, Norway, Latvia, the United Kingdom, Czech Republic, Bangladesh, and Australia).	A randomized, double-blind, placebo-controlled trial.	Influenza vaccine vs. placebo	12 months	• **Study focus:** Patients with recent myocardial infarction or high-risk coronary heart disease enrolled across 30 centers in 8 countries. • In the modified intention-to-treat population (*n* = 2532), the primary composite outcome occurred less often with vaccination than placebo (5.3% vs. 7.2%; HR 0.72; 95% CI 0.52–0.99; *p* = 0.040). • All-cause mortality was lower in the vaccine group (2.9% vs. 4.9%). • Cardiovascular mortality was also lower in the vaccine group (2.7% vs. 4.5%). • No significant reduction was observed for MI alone (2.0% vs. 2.4%).	Early influenza vaccination after myocardial infarction or in patients with high-risk coronary heart disease was associated with a lower risk of the composite outcome of all-cause mortality, myocardial infarction, or stent thrombosis, as well as lower all-cause and cardiovascular mortality at 12 months compared with placebo.
**Gurfinkel 2004** **[14]**	Argentina	A randomized, prospective, multicenter, parallel-group, controlled pilot study	Influenza vaccine vs. control	12 months	• **Study focus:** Patients with acute coronary syndromes and those undergoing planned percutaneous coronary intervention. • The composite outcome occurred less often in vaccinated patients than controls (22% vs. 37%; HR 0.59; 95% CI 0.40–0.86; *p* = 0.004). • Vaccination was associated with lower overall event risk (RR 0.34; 95% CI 0.17–0.71; *p* = 0.002). • The greatest benefit was observed in patients with acute myocardial infarction. • Greater benefit was also noted in patients with higher TIMI risk scores and non-ST-segment deviation.	Influenza immunization may reduce death and ischemic events in patients with myocardial infarction and those undergoing angioplasty during influenza season. The 1-year follow-up supports this benefit, although larger confirmatory trials are still needed.
**Akhtar 2023** **[12]** *	Australia, Bangladesh, Czech Republic, Latvia, Denmark, Norway, Sweden, and the UK.	A randomized, double-blind, placebo-controlled, investigator-initiated trial	Influenza vaccine vs. placebo	12 months	• **Study focus:** Secondary timing analysis of patients with acute myocardial infarction from the IAMI trial, comparing earlier versus later vaccination. • Early and late vaccination showed no significant difference in efficacy for adverse cardiovascular events. • Vaccine efficacy estimates were similar for all-cause and cardiovascular death across timing groups. • All-cause mortality was numerically lower in the early-vaccination group, but the interaction was not statistically significant. • Timing of vaccination did not significantly modify clinical benefit.	The trial showed no significant difference in efficacy between early and late influenza immunization; however, the findings support influenza vaccination in patients with cardiovascular disease.
**Phrommintikul 2010** **[11]**	Thailand	A prospective, randomized, open-label trial with blinded endpoint assessment (PROBE design)	Inactivated influenza vaccine in the vaccine group and no treatment in the control group	12 months	• **Study focus:** Patients with acute coronary syndrome receiving inactivated influenza vaccine versus no treatment. • Major cardiovascular events occurred less often in vaccinated patients than controls (9.5% vs. 19.3%; *p* = 0.004). • Vaccination was associated with lower risk of the primary composite endpoint (HR 0.70; 95% CI 0.57–0.86). • Cardiovascular death was numerically lower in the vaccine group, but the difference was not statistically significant.	Influenza vaccination reduced major cardiovascular events in patients with acute coronary syndrome and should be encouraged as a secondary preventive strategy in this population.
**Dehesh 2025** **[24]**	Iran	A randomized, placebo-controlled, single-blind clinical trial	Influenza vaccine vs. placebo	12 months	• **Study focus:** Patients with coronary artery disease randomized to influenza vaccine or placebo. • Cardiovascular mortality was similar between groups (2.29% vs. 2.22%). • All-cause mortality was also similar between groups (3.05% vs. 2.96%). • ACS, MI, and coronary revascularization were numerically lower with vaccination, but none reached statistical significance. • Antibody responses were higher after vaccination, with no correlation to cardiovascular outcomes.	Influenza vaccination may improve cardiovascular outcomes, although this potential benefit was not correlated with post-vaccination antibody titers.
**Loeb 2022** **[16]**	30 centers (mostly hospitals affiliated with universities or a research institute) in ten countries in Asia, the Middle East, and Africa (7 in India, 4 in the Philippines, 4 in Nigeria, 6 in China, 1 in Zambia, 2 in Mozambique, 3 in Saudi Arabia, 1 in Kenya, 1 in Uganda, and 1 in the United Arab Emirates).	A multinational, randomized, double-blind, placebo-controlled trial	Influenza vaccine vs. placebo	3 years	• **Study focus:** Patients with heart failure enrolled in a multinational placebo-controlled trial across Asia, the Middle East, and Africa. • During overall follow-up, neither co-primary cardiovascular outcome was significantly reduced with vaccination. • Vaccination was associated with lower all-cause hospitalization and lower pneumonia rates. • No significant difference was observed for all-cause death, cardiovascular death, nonfatal MI, stroke, or heart failure hospitalization. • During peak influenza circulation, some cardiovascular and mortality benefits were observed.	Although the pre-specified co-primary outcomes over the full observation period were not statistically significant, the reduction during peak influenza circulation suggests a possible clinical benefit, particularly given the reduction in pneumonia, hospitalizations, and some cardiovascular events.
**Anderson 2025** **[25]**	China	A multiregional, two-arm, parallel-group, open-label, multiple-period, cluster-randomized controlled trial	Influenza vaccination vs. usual care	12 months	• **Study focus:** Hospitalized patients with acute heart failure enrolled in a multiregional cluster-randomized trial in China. • The primary outcome occurred less often with vaccination than usual care (41.2% vs. 47.0%; OR 0.83; 95% CI 0.72–0.97; *p* = 0.019). • Results remained consistent in sensitivity analyses. • Major adverse events were also less frequent in the vaccination group. • Vaccination during hospitalization was associated with improved 1-year outcomes.	Influenza immunization during hospitalization for acute heart failure may improve survival and reduce readmissions over the following year. Inpatient vaccination may be a practical strategy for high-risk patients in both resource-limited and resource-rich settings.

*: Akhtar 2023 represents a secondary analysis of the IAMI trial population. Abbreviations: ACS, acute coronary syndrome; HR, hazard ratio; MACEs, major adverse cardiovascular events; MI, myocardial infarction; PCI, percutaneous coronary intervention; PROBE, prospective randomized open, blinded endpoint; TIMI, Thrombolysis in Myocardial Infarction. Note: Effect estimates are reported as presented in the original studies.

**Table 2:** The baseline characteristics of the included studies.

ID	Groups	Sample Size	Age, Years *	Males, n (%)	BMI, Kg/m^2^ *
**Ciszewski 2010** **[15]**	ACS-primary PCI	157	58.5 (50.9–69.1) †	106 (67.5%)	27.2 (24.9–30.8) †
	Stable angina	501	58.3 (51.0–67.3) †	371 (74.5%)	27.9 (25.5–30.4) †
**Fröbert 2021** **[13]**	Influenza vaccine	1272	60.1 (11.0)	1036 (81.4%)	27.5 (5.0)
	Placebo	1260	59.6 (11.4)	1034 (82.1%)	27.4 (5.1)
**Gurfinkel 2004** **[14]**	Influenza vaccine	100	64	NR	NR
	Control	100	66	NR	NR
**Akhtar 2023** **[12]**	Influenza vaccine	1272	59.9 (11.2) ‡	2070 (81.75%) ‡	27.5 (5.0)
	Placebo	1260			
**Phrommintikul 2010** **[11]**	Influenza vaccine	221	65 (9)	135 (61%)	NR
	Control	218	67 (9)	114 (52%)	NR
**Dehesh 2025** **[24]**	Influenza vaccine	137	54.53 (9.21)	92 (67.2%)	27.63 (4.48)
	Placebo	141	54.93 (8.98)	93 (66%)	27.75 (4.48)
**Loeb 2022** **[16]**	Influenza vaccine	2560	57.4 (15.1)	1227 (47.9%)	NR
	Placebo	2569	57.0 (15.6)	1264 (49.2%)	NR
**Anderson 2025** **[25]**	Influenza vaccine	3570	71.7 (11.0)	1888 (52.9%)	23.9 (4.0)
	Usual care	4201	72.0 (11.5)	2218 (52.8%)	24.0 (4.2)

*: Data are presented as mean (SD); †: Data are represented as Median (IQR); ‡: Data are presented for the overall study cohorts. Abbreviations: ACS, acute coronary syndrome; BMI, body mass index; PCI, percutaneous coronary intervention; NR, not reported.

**Table 3:** Certainty of evidence.

Outcome	Certainty of Evidence	Relative Effect (95% CI)	No. of Participants (Studies)	Comments
Composite of all-cause death, AMI, or stent thrombosis	**Moderate (** **⊕⊕⊕** **◯)**	OR 0.71 (0.57 to 0.90)	5064 (5 studies)	Downgraded one level for risk of bias.
MACEs (cardiovascular death, MI, or coronary revascularization)	**Moderate (** **⊕⊕⊕** **◯)**	OR 0.44 (0.26 to 0.74)	904 (2 studies)	Downgraded one level for risk of bias.
Cardiovascular death	**Moderate (** **⊕⊕⊕** **◯)**	OR 0.64 (0.47 to 0.86)	11,703 (8 studies)	Downgraded one level for risk of bias; not downgraded for inconsistency because heterogeneity was only moderate and the direction of effect was consistent across studies; not downgraded for imprecision because the 95% CI excluded the line of no effect.
Myocardial infarction	**Low (** **⊕⊕** **◯◯)**	OR 0.82 (0.57 to 1.18)	8633 (5 studies)	Downgraded one level for risk of bias and one level for imprecision because the confidence interval crossed the line of no effect.
All-cause mortality	**Very low (** **⊕** **◯◯◯)**	OR 1.13 (0.79 to 1.62)	17,732 (6 studies)	Downgraded one level for risk of bias, one level for inconsistency (substantial heterogeneity, I^2^ = 88%), and one level for imprecision because the confidence interval was wide and crossed the line of no effect.

Abbreviations: AMI, acute myocardial infarction; CI, confidence interval; MACEs, major adverse cardiovascular events; MI, myocardial infarction; OR, odds ratio. Certainty of evidence was assessed using the GRADE approach: Moderate, ⊕⊕⊕◯; Low, ⊕⊕◯◯; Very low, ⊕◯◯◯.

## Data Availability

The data that support the findings of this study are available from the corresponding author upon reasonable request.

## Abbreviations

The following abbreviations are used in this manuscript:

MDPI	Multidisciplinary Digital Publishing Institute
DOAJ	Directory of Open Access Journals
TLA	Three-letter acronym
LD	Linear dichroism
CAD	Coronary artery disease

## References

[1] Roth G.A., Mensah G.A., Fuster V. (2020). The Global Burden of Cardiovascular Diseases and Risks: A Compass for Global Action. J. Am. Coll. Cardiol..

[2] Tsao C.W., Aday A.W., Almarzooq Z.I., Anderson C.A.M., Arora P., Avery C.L., Baker-Smith C.M., Beaton A.Z., Boehme A.K., Buxton A.E. (2023). Heart Disease and Stroke Statistics-2023 Update: A Report From the American Heart Association. Circulation.

[3] Kwong J.C., Schwartz K.L., Campitelli M.A., Chung H., Crowcroft N.S., Karnauchow T., Katz K., Ko D.T., McGeer A.J., McNally D. (2018). Acute Myocardial Infarction after Laboratory-Confirmed Influenza Infection. N. Engl. J. Med..

[4] Udell J.A., Zawi R., Bhatt D.L., Keshtkar-Jahromi M., Gaughran F., Phrommintikul A., Ciszewski A., Vakili H., Hoffman E.B., Farkouh M.E. (2013). Association between Influenza Vaccination and Cardiovascular Outcomes in High-Risk Patients: A Meta-Analysis. JAMA.

[5] Warren-Gash C., Smeeth L., Hayward A.C. (2009). Influenza as a Trigger for Acute Myocardial Infarction or Death from Cardio-vascular Disease: A Systematic Review. Lancet Infect. Dis..

[6] Yousif K.A. (2025). Seasonal Influenza: A Narrative Review of Epidemiology, Clinical Features, and Preventive Strategies. Cureus.

[7] Lian R., Zhang H., An Y., Chen Z. (2025). Chronic Diseases and Influenza Vaccines. Vaccines.

[8] Heidecker B., Libby P., Vassiliou V.S., Roubille F., Vardeny O., Hassager C., Gatzoulis M.A., Mamas M.A., Cooper L.T., Schoenrath F. (2025). Vaccination as a New Form of Cardiovascular Prevention: A European Society of Cardiology Clinical Consensus Statement. Eur. Heart J..

[9] Chow J.K., Bansal N. (2026). ISHLT Statement on Vaccines in Transplant Recipients. J. Heart Lung Transpl..

[10] Yedlapati S.H., Khan S.U., Talluri S., Lone A.N., Khan M.Z., Khan M.S., Navar A.M., Gulati M., Johnson H., Baum S. (2021). Effects of Influenza Vaccine on Mortality and Cardiovascular Outcomes in Patients with Cardiovascular Disease: A Sys-tematic Review and Meta-Analysis. J. Am. Heart Assoc..

[11] Phrommintikul A., Kuanprasert S., Wongcharoen W., Kanjanavanit R., Chaiwarith R., Sukonthasarn A. (2011). Influenza Vac-cination Reduces Cardiovascular Events in Patients with Acute Coronary Syndrome. Eur. Heart J..

[12] Akhtar Z., Götberg M., Erlinge D., Christiansen E.H., Oldroyd K.G., Motovska Z., Erglis A., Hlinomaz O., Jakobsen L., Engstrøm T. (2023). Optimal Timing of Influenza Vaccination among Patients with Acute Myocardial Infarction—Findings from the IAMI Trial. Vaccine.

[13] Fröbert O., Götberg M., Erlinge D., Akhtar Z., Christiansen E.H., MacIntyre C.R., Oldroyd K.G., Motovska Z., Erglis A., Moer R. (2021). Influenza Vaccination After Myocardial Infarction: A Randomized, Double-Blind, Placebo-Controlled, Multi-center Trial. Circulation.

[14] Gurfinkel E.P., Leon de la Fuente R., Mendiz O., Mautner B. (2004). Flu Vaccination in Acute Coronary Syndromes and Planned Percutaneous Coronary Interventions (FLUVACS) Study. Eur. Heart J..

[15] Ciszewski A., Bilińska Z.T., Kępka C., Kruk M., Księżycka-Majczyńska E., Rużyłło W. (2010). The Protective Effect of Influenza Vaccination on the Clinical Course of Coronary Disease in Patients with Acute Coronary Syndromes Treated by Primary PCI—A Report from FLUCAD Study. Adv. Interv. Cardiol..

[16] Loeb M., Roy A., Dokainish H., Dans A., Palileo-Villanueva L.M., Karaye K., Zhu J., Liang Y., Goma F., Damasceno A. (2022). Influenza Vaccine to Reduce Adverse Vascular Events in Patients with Heart Failure: A Multinational Randomised, Dou-ble-Blind, Placebo-Controlled Trial. Lancet Glob. Health.

[17] Christensen J., Johansen N.D., Modin D., Janstrup K.H., Nealon J., Samson S., Loiacono M., Harris R., Larsen C.S., Jensen A.M.R. (2025). Relative Effectiveness of High-Dose Versus Standard-Dose Quadrivalent Influenza Vaccine in Older Adults with Cardiovascular Disease: A Prespecified Analysis of the DANFLU-1 Randomized Clinical Trial. Circ. Cardiovasc. Qual. Outcomes.

[18] Loomba R.S., Aggarwal S., Shah P.H., Arora R.R. (2012). Influenza Vaccination and Cardiovascular Morbidity and Mortality: Analysis of 292,383 Patients. J. Cardiovasc. Pharmacol. Ther..

[19] LeBras M.H., Barry A.R. (2017). Influenza Vaccination for Secondary Prevention of Cardiovascular Events: A Systematic Review. Can. J. Hosp. Pharm..

[20] Bhugra P., Grandhi G.R., Mszar R., Satish P., Singh R., Blaha M., Blankstein R., Virani S.S., Cainzos-Achirica M., Nasir K. (2021). Determinants of Influenza Vaccine Uptake in Patients with Cardiovascular Disease and Strategies for Improvement. J. Am. Heart Assoc..

[21] Zangiabadian M., Nejadghaderi S.A., Mirsaeidi M., Hajikhani B., Goudarzi M., Goudarzi H., Mardani M., Nasiri M.J. (2020). Protective Effect of Influenza Vaccination on Cardiovascular Diseases: A Systematic Review and Meta-Analysis. Sci. Rep..

[22] Page M.J., McKenzie J.E., Bossuyt P.M., Boutron I., Hoffmann T.C., Mulrow C.D., Shamseer L., Tetzlaff J.M., Akl E.A., Brennan S.E. (2021). The PRISMA 2020 Statement: An Updated Guideline for Reporting Systematic Reviews. BMJ.

[23] Cochrane Collaboration (2020). RevMan.

[24] Dehesh M., Gholamin S., Razavi S.-M., Eskandari A., Vakili H., Rahnavardi Azari M., Wang Y., Gough E.K., Keshtkar-Jahromi M. (2025). Influenza Vaccination and Cardiovascular Outcomes in Patients with Coronary Artery Diseases: A Placebo-Controlled Randomized Study, IVCAD. Vaccines.

[25] Anderson C.S., Hua C., Wang Z., Wang C., Jiang C., Liu R., Han R., Li Q., Shan S., Billot L. (2025). Influenza Vaccination to Improve Outcomes for Patients with Acute Heart Failure (PANDA II): A Multiregional, Seasonal, Hospital-Based, Clus-ter-Randomised, Controlled Trial in China. Lancet.

[26] Nguyen J.L., Yang W., Ito K., Matte T.D., Shaman J., Kinney P.L. (2016). Seasonal Influenza Infections and Cardiovascular Disease Mortality. JAMA Cardiol..

[27] Kodaira M., Hasan M.S., Grossman Y., Guerrero C., Guo L., Liu A., Therrien J., Marelli A. (2024). Risk of Cardiovascular Events after Influenza Infection-Related Hospitalizations in Adults with Congenital Heart Disease: A Nationwide Population Based Study. Am. Heart J..

[28] Di Cesare M., Perel P., Taylor S., Kabudula C., Bixby H., Gaziano T.A., McGhie D.V., Mwangi J., Pervan B., Narula J. (2024). The Heart of the World. Glob. Heart.

[29] (2025). Global Burden of Cardiovascular Diseases and Risks 2023 Collaborators. Global, Regional, and National Burden of Cardiovascular Diseases and Risk Factors in 204 Countries and Territories, 1990–2023. J. Am. Coll. Cardiol..

[30] Behrouzi B., Bhatt D.L., Cannon C.P., Vardeny O., Lee D.S., Solomon S.D., Udell J.A. (2022). Association of Influenza Vaccination with Cardiovascular Risk: A Meta-Analysis. JAMA Netw. Open.

[31] Jaiswal V., Ang S.P., Yaqoob S., Ishak A., Chia J.E., Nasir Y.M., Anjum Z., Alraies M.C., Jaiswal A., Biswas M. (2022). Cardio-protective Effects of Influenza Vaccination among Patients with Established Cardiovascular Disease or at High Cardiovascular Risk: A Systematic Review and Meta-Analysis. Eur. J. Prev. Cardiol..

[32] Liu X., Zhang J., Liu F., Wu Y., Li L., Fan R., Fang C., Huang J., Zhang D., Yu P. (2025). Association between Influenza Vaccination and Prognosis in Patients with Ischemic Heart Disease: A Systematic Review and Meta-Analysis of Randomized Controlled Trials. Travel. Med. Infect. Dis..

[33] Liu R., Fan Y., Patel A., Liu H., Du X., Liu B., Di Tanna G.L. (2024). The Association between Influenza Vaccination, Cardiovas-cular Mortality and Hospitalization: A Living Systematic Review and Prospective Meta-Analysis. Vaccine.

[34] Vardeny O., Kim K., Udell J.A., Joseph J., Desai A.S., Farkouh M.E., Hegde S.M., Hernandez A.F., McGeer A., Talbot H.K. (2021). Effect of High-Dose Trivalent vs Standard-Dose Quadrivalent Influenza Vaccine on Mortality or Cardiopulmonary Hospitalization in Patients with High-Risk Cardiovascular Disease: A Randomized Clinical Trial. JAMA.

[35] Lassen M.C.H., Johansen N.D., Modin D., Nealon J., Samson S., Dufournet M., Loiacono M.M., Larsen C.S., Jensen A.M.R., Landler N.E. (2024). Effects of High-Dose versus Standard-Dose Quadrivalent Influenza Vaccine among Patients with Diabetes: A Post-Hoc Analysis of the DANFLU-1 Trial. Diabetes Obes. Metab..

[36] Alhabeeb W., Elasfar A., Kinsara A.J., Aljizeeri A., Jelaidan I., Alghalayini K., AlKheraiji M.F., Akbar M., Lawand S., Alyousif S.M. (2024). A Saudi Heart Association Position Statement on Cardiovascular Diseases and Diabetes Mellitus. J. Saudi Heart Assoc..

[37] Al-Awaidy S.T., Al Raisi S.S., Al Slail F., Al Kathiry D.A., Al Mutairy A.O., Jumah I.M.B., Fadl S.M., Abdelfadil K.O., Kheir O., Koul P.A. (2024). Mitigating the Burden of Seasonal Influenza on Cardiovascular Diseases in GCC Countries. Oman Med. J..

